# Integrating Consumer-Grade Wearable Devices and Patient-Generated Health Data into Clinical Care: Perspectives from Healthcare Professionals at a Learning Health System

**DOI:** 10.1007/s11606-025-09876-x

**Published:** 2025-10-09

**Authors:** Selene S. Mak, Rebecca L. Kinney, Anne L. Bailey, Allison E. Gaffey, Mark R. Relyea, Elias K. Spanakis, Garrett I. Ash

**Affiliations:** 1 Veterans Health Administration at the Greater Los Angeles Healthcare System, Los Angeles, CA USA; 2 VA Center for the Study of Healthcare Innovation, Implementation & Policy (CSHIIP), Los Angeles, CA USA; 3 Department of Population and Quantitative Health Sciences, Division of Preventive and Behavioral Medicine, University of Massachusetts Chan Medical School, Worcester, MA USA; 4 U.S. Department of Veterans Affairs, VA National Center On Homelessness Among Veterans, Washington, DC USA; 5 Department of Veteran Affairs, Veterans Health Administration Strategic Initiatives Lab, Washington, DC USA; 6 Department of Internal Medicine, Section of Cardiovascular Medicine, Yale School of Medicine, New Haven, CT USA; 7 Veterans Affairs Connecticut Healthcare System, West Haven, CT USA; 8 Department of Psychiatry, Yale School of Medicine, New Haven, CT USA; 9 Division of Endocrinology, Baltimore Veterans Affairs Medical Center, Baltimore, MD USA; 10 Division of Endocrinology, Diabetes, and Nutrition, University of Maryland School of Medicine, Baltimore, MD USA; 11 Department of Internal Medicine, Section of General Internal Medicine, Yale School of Medicine, New Haven, CT USA; 12 Department of Biomedical Informatics and Data Science, Yale School of Medicine, New Haven, CT USA; 13 Pain, Research, Informatics, Medical Comorbidities and Education Center, Vetrans Affairs Connecticut Healthcare System, West Haven, CT USA

**Keywords:** digital health, patient-generated health data, clinical workflow, learning health system

## Abstract

**Background:**

The increasing popularity of consumer-grade wearable (CGW) devices for everyday use has prompted discussions within learning healthcare systems (LHSs) about the integration of patient-generated health data (PGHD) into clinical workflow.

**Objective:**

To report findings from interviews with healthcare professionals (HCPs) about the potential role of CGWs and PGHD in clinical workflow, which in turn informed recommendations for health systems to consider when integrating PGHD from CGWs.

**Design:**

Mixed methods.

**Participants:**

Clinical care providers and holistic/wellness providers with various training and expertise from the largest integrated health system in the USA (i.e., Veterans Health Administration).

**Approach:**

A purposive sample of 29 individuals who were knowledgeable informants were invited to complete a survey focused on CGW feasibility and usability of PGHD. Those who completed the survey were invited to participate in a semi-structured interview to elicit their perceptions of the barriers and facilitators for PGHD utilization. Survey data were analyzed descriptively and rapid qualitative analysis techniques were used to identify themes from interview data.

**Key Results:**

Twenty-one HCPs were included in this study. Quantitative and qualitative findings revealed significant interest in PGHD integration in clinical workflow and patient interactions. Clinical care providers focused on the value of PGHD as a complement to traditional clinical data, whereas holistic/wellness providers emphasized using CGWs and PGHD for behavioral change. Their interest levels were tempered by concerns about workload implications and apprehension about having to distill the vast amount of PGHD of varying validity into clinically actionable information.

**Conclusions:**

Integrating CGWs and PGHD can enable an LHS to improve patient outcomes, particularly to promote health access and equity. Yet, concerns were raised regarding staffing and workload issues, data interpretation, expectations, access, privacy, and educational needs. Therefore, harnessing the benefits of CGWs will require investing in health systems’ infrastructure to optimally integrate PGHD into clinical workflow.

**Supplementary Information:**

The online version contains supplementary material available at https://doi.org/10.1007/s11606-025-09876-x.

## INTRODUCTION

Patients’ evolving health needs, a heightened interest in consumer-grade wearable (CGW) devices, and changes in the ways patients manage their health and seek care have prompted crucial discussions across many healthcare systems on how to integrate CGWs and their data into clinical workflows. As of 2023, almost half of US adults own at least one wearable device.^1^ Rapid adoption of devices and applications has accelerated the collection of patient-generated health data (PGHD), which is health-related information that individuals create, record, or gather outside of a clinical encounter and facilitates the monitoring of one’s health.^2^ Examples of PGHD collected by CGWs include step count and heart rate. CGWs and PGHD are emerging clinical advancements with the potential to impact health outcomes and treatment.^3^ Healthcare professionals (HCPs) from various health systems report that PGHD may improve clinical decisions,^4^ “connected health” between patients and their care teams,^5–8^ and can help individuals understand their health and treatment options.^9^

The utility of PGHD generated from CGWs is of particular interest to learning health systems (LHS), given that data facilitates the continuous improvement of patient-centric, healthcare practice.^10–12^ A health system becomes an LHS when it has the socio-technical infrastructure—people, technology, processes, and policies—to support data collection and analysis and routinely applies data-driven insights to its continuous improvement programs.^10,13,14^ Such an LHS is then shaped and reshaped by tackling challenges, such as complex health problems, changing policies, innovative healthcare delivery, and the shifting dynamics of human behavior.^10,15^ Still, it remains unclear how to integrate CGWs and PGHD into clinical workflow to enhance an LHS’s efforts for continuous quality improvement, while also resolving ongoing challenges.

Despite the growing use of CGWs, there remains a lack of standardized protocols and clinical guidelines for their integration into practice. Without clinical guidance, HCPs are forced to independently determine the appropriate use of CGWs and the protocol for integrating PGHD into clinical workflow. Additionally, unlike clinical-grade remote monitoring tools, CGWs generate data of varying reliability, necessitating careful consideration of their role in clinical decision-making. This mixed methods study explored HCPs’ attitudes and perspectives on the potential role and utility of CGWs and PGHD in clinical workflow, and reported barriers and opportunities associated with CGWs and PGHD, and recommendations for the integration of CGWs and PGHD into clinical workflow.

## METHODS

### Overview

This study was conducted in 16 Veterans Health Administration (VHA) facilities. The VHA is the largest integrated healthcare system in the USA with 172 medical centers and 1138 outpatient sites serving approximately nine million Veterans of the United States Armed Forces.^16^ This study was considered quality improvement and, thus, did not require research oversight by the local Institutional Review Board. The Consolidated Criteria for Reporting Qualitative Research (COREQ) guidelines were used (Appendix I).^17^

### Participant Selection

Veterans Health Affairs Innovation Ecosystem (VHAIE), part of the VHA Office of Healthcare Innovation and Learning, had recruited a national cohort of VHA HCPs to serve as facility leads to distribute Fitbit™ devices to Veterans.^18,19^ VHAIE provided us with emails of all HCPs who had participated in the distribution program (*n* = 29). Of these, three individuals had left the VA. An invitation to participate in this study was emailed to the remaining 26 individuals on this list and included a link to the survey. Microsoft Teams direct messaging application was used to follow-up with individuals who did not respond to the email. Three HCPs who were not on VHAIE’s list received the email invitation because their colleague mentioned these individuals also served as facility leads. Thus, overall, a purposive sample of 29 eligible individuals was identified and invited. Of these, one individual did not respond to the email invitation, and two declined to complete the survey. A total of 26 participants who had completed the survey were then invited to an interview, of which two did not respond to the interview invitation, and one declined to participate. Participants were not compensated for their participation in this study.

### Procedure

The study was conducted in two phases: (1) a quantitative survey to capture participants’ perspectives on the feasibility of and interest in using CGWs with patients, and (2) a semi-structured interview to gain insights into participants’ perspectives and experiences with CGWs in clinical workflow. Respondents who completed the survey were invited to participate in the interview. Those who verbally consented were then interviewed.

#### Quantitative Survey

The survey was adapted from a survey designed for physiotherapists to share their perspectives on using mobile or wearable technology in their practices.^20^ Questions concerned participants’ demographics (e.g., age, education, job role) and perspectives on the feasibility of using CGWs (6-point Likert items). The survey was programmed in Qualtrics (https://www.qualtrics.com).

#### Semi-structured Interviews

The research team developed the semi-structured interview guide with input from VHAIE (Appendix II). The guide was constructed as a series of open-ended questions about (1) experiences with CGWs in clinical care; (2) barriers and opportunities for CGWs; and (3) goals and vision to integrate CGWs into clinical workflow. Each interview ended with an invitation to brainstorm characteristics or features of an ideal program to integrate CGWs and PGHD into clinical workflow with the question, “Suppose you were ruler for the day and could make all the changes you needed to set up a program to integrate patient-generated data into clinical workflow. What would that look like today?”

Interviews were conducted using Microsoft Teams in April–June 2022 and facilitated by two PhD-level researchers with expertise in digital health technologies and experienced in qualitative methodologies (SM, female; GA, male). Interviewers did not have a prior relationship with any of the interviewees. At the beginning of each interview, the interviewer introduced themselves and restated the purpose of the interview. Interviews were audio recorded and averaged around 30 min in length. Field notes were completed after each interview.

### Data Analysis and Synthesis

The sample was subdivided between clinical care providers and holistic/wellness providers since the latter does not provide clinical assessment or prescribe treatment, which could influence their perceived usefulness of CGWs and PGHD in a clinical setting. Survey data was tabulated and analyzed according to standard descriptive approaches for Likert-type interval data, including mean and standard deviation of raw responses and proportion giving a favorable response. The open-ended survey question and interview transcripts from Microsoft Teams were analyzed using rapid qualitative analysis techniques to identify themes. An advantage of employing rapid qualitative analysis is enhanced efficiency in data collection and analysis to broaden our understanding of an applied policy or research question.^21^ While the research team did develop a priori categories and codes based on the literature and the interview guide, an inductive approach was still utilized, allowing the data to determine the main themes.

The interview transcripts were read by the two team members (SM, GA) who identified and summarized codes that were used to generate themes within each of the broader categories of the interview. As themes emerged from the data, new codes were added and grouped by themes evident across the interviews. A template with these themes were created, and each transcript was independently summarized by one researcher in Microsoft Word and reviewed by the other. Summaries were consolidated into a matrix and domains were grouped into themes for further synthesis.^22,23^ The two researchers met weekly to discuss and resolve discrepancies in interpretation of data. Selected quotes are presented alongside overarching themes, and responses to one of the questions are mapped to the four enabling features of an LHS from the Learning Health System Consolidated Framework (LHS-CF).^24^

## RESULTS

Twenty-three individuals completed the survey and interview. Data from two participants were not included in our analyses because their job role (administrators) did not involve patient care, leaving a sample of 21 HCPs. Those who provided patient clinical care were defined as “clinical care providers” (i.e., physicians, nurses, dietitians, social workers), and HCPs who performed supplemental patient care were designated “holistic/wellness care providers” (i.e., wellness coaches, recreational therapist).

### Participant Characteristics

Of the 21 participants, 57.1% (*n* = 12) were female, 90.5% (*n* = 19) were < 55 years old, < 80% non-Hispanic White, and 80.9% (*n* = 17) owned at least one CGW (Table 1). Most were clinical care providers and reported working in their profession for more than 10 years. Nearly all (95.2%, *n* = 20) reported using CGWs in patient care at least some of the time; approximately one-quarter (23.5%, *n* = 5) reported using them most of the time.

**Table 1:** Characteristics of VA Clinical Care Providers and Holistic/Wellness Providers (*n* = 21)

Demographics	Total *N* (%)	Holistic/wellness providers (*n* = 6) *N* (%)	Clinical care providers (*n* = 15) *N* (%)
Sex			
Female	12 (57.1)	4 (66.7)	8 (53.3)
Male	8 (38.1)	2 (33.3)	6 (40.0)
Preferred not to say	1 (4.8)	0 (0.0)	1 (6.7)
Age range (years)			
25–34	1 (4.8)	0	1 (6.7)
35–44	9 (42.9)	1 (16.7)	8 (43.3)
45–54	6 (28.6)	2 (33.3)	4 (26.7)
55 +	5 (23.8)	3 (66.7)	2 (13.3)
Education			
MD, including MD with PhD or MS	6 (28.6)	0 (0.0)	6 (40.0)
DO, including DO with MPH	2 (9.5)	0 (0.0)	2 (13.3)
PsyD	1 (4.8)	0 (0.0)	1 (6.7)
MSW/BSW	3 (14.3)	1 (16.7)	2 (13.3)
MSN/BSN	3 (14.3)	1 (16.7)	2 (13.3)
MS	2 (9.5)	0 (0.0)	2 (13.3)
BA/other	4 (19.0)	4 (66.7)	0 (0.0)
Area of specialization			
Adaptive Sports and Fitness	1 (4.8)	1 (16.7)	0 (0.0)
Clinical Psychology	1 (4.8)	0 (0.0)	1 (6.7)
Family Medicine	1 (4.8)	0 (0.0)	1 (6.7)
Health Coaching	5 (23.8)	5 (83.3)	0 (0.0)
Internal Medicine	3 (14.3)	0 (0.0)	3 (20.0)
Nutrition and Dietetics	2 (9.5)	0 (0.0)	2 (13.3)
Occupational Health Nursing	1 (4.8)	0 (0.0)	1 (6.7)
Preventive Medicine	1 (4.8)	0 (0.0)	1 (6.7)
Psychiatry	2 (9.5)	0 (0.0)	2 (13.3)
Sleep Medicine	2 (9.5)	0 (0.0)	2 (13.3)
Suicide Prevention/Postvention	1 (4.8)	0 (0.0)	1 (6.7)
Telehealth Nursing	1 (4.8)	0 (0.0)	1 (6.7)
Job role			
Physician (MD/DO)			9 (60.0)
Nurse			3 (20.0)
Dietitian			2 (13.3)
Social worker			1 (6.7)
Health coach		5 (83.3)	
Recreational therapist		1 (16.7)	
Years in profession			
< 5 years	4 (19.0)	2 (33.3)	2 (13.3)
5–10 years	3 (14.3)	0 (0.0)	3 (20.0)
11–20 years	7 (33.3)	3 (50.0)	4 (26.7)
> 20 years	7 (33.3)	1 (16.7)	6 (40.0)
Personal device ownership			
At least one device	17 (80.9)	5 (83.3)	12 (80.0)
None	4 (19.0)	1 (16.7)	3 (20.0)
Use of CGWs in patient care			
All of the time	1 (4.8)	1 (16.7)	0 (0.0)
Most of the time	5 (23.8)	2 (33.3)	3 (20.0)
Half of the time	2 (9.5)	1 (16.7)	1 (6.7)
Some of the time	12 (57.1)	1 (16.7)	11 (73.3)
Not at all	1 (4.8)	1 (16.7)	0 (0.0)

### Survey

Respondents reported a high level of interest in using CGWs to promote engagement between patients and HCPs (Table 2; Appendix V presents data by job role). However, HCPs were slightly less interested in monitoring patient progress (81.0%, *n* = 17). The majority (81.0%; *n* = 17) suggested using CGWs in patient care would be feasible with adequate support. When asked to elaborate on “adequate support,” respondents mentioned several ideas that included technical support for patients, such as a digital navigator, and improved methods to share data smoothly and securely.

**Table 2:** Survey Responses of VHA Clinical Care and Holistic (physicians, nurses, dietitians, social workers)/Wellness Providers (wellness coaches and recreational therapist) (*n* = 21)

Survey items	Agree or strongly agree		Mean (SD) out of 5
**Interest in uses of CGWs at clinical encounters** (1 = strongly disagree, 2 = disagree, 3 = neither agree or disagree, 4 = agree, 5 = strongly agree)			
CGWs can promote engagement between the health care provider and patient or caregiver	19 (90.5)	4.1 (0.6)	
In the future, if my patient wore a sensor that automatically collected information about their health or well-being, I would refer to the data to understand how my patient is responding to treatment	19 (90.5)	4.4 (0.7)	
I would be willing to try out a new CGW technology in my practice before it had been clinically validated	18 (85.7)	4.3 (0.7)	
CGWs may provide me with a way to communicate my patient’s progress more clearly	18 (85.7)	4.2 (0.7)	
I would find it useful to use CGWs to monitor my patient’s progress (e.g., using a Fitbit to assess their daily activity levels)	17 (81.0)	4.2 (0.8)	
**Interest in CGWs outside of clinical encounters**			
Consumer-grade wearable devices may help my patients stay engaged (especially outside our face-to-face sessions)	20 (95.2)	4.3 (0.6)	
**Interest in add-ins**			
I would like consumer-grade wearable devices to interface with mobile video- and text-based coaching	18 (85.7)	4.4 (0.7)	
I would like consumer-grade wearable devices to interface with mobile instructional videos (e.g., Fitbit Premium)	19 (90.5)	4.3 (0.6)	
**Feasibility**			
I expect learning how to use a CGW in a way that helps my patients will be quite difficult for me	2 (10.0)	2.1 (1.0)	
I expect it will be difficult to teach or coach my patients on the use of a CGW (e.g., Fitbit, Oura Ring™)	4 (19.0)	2.6 (0.9)	
I expect using a CGW in my practice will take a lot of extra time	3 (14.3)	2.8 (1.0)	
I would be willing to use a new CGW technology if I received adequate support	17 (81.0)	4.2 (0.7)	
Please elaborate on adequate support (Open response): • Technical support for patients • Digital navigators • Active follow-up to prevent patients from passively abandoning device • Improved methods to share data smoothly and securely • Digital technology coach to train patients • A direct help line for patients • Clearly written user guide • Online platform to ask questions • Customer service education • Troubleshooting assistance to offload level of help needed from the institution • Tech support line • YouTube video tech support for patients • Live patient level support			

The frequency of agreed/strongly agreed survey responses is presented as *n* (%)

### Semi-structured Interviews

Three themes emerged from our analysis, including (1) use and utility of CGWs and PGDH, (2) barriers to utilizing CGWs and PGHD, and (3) suggestions for integration of CGWs and PGHD into patient care. Selected participant quotes are presented below; see Appendix III for additional quotes by theme.

#### Theme 1: Use and Utility of CGWs and PGHD

##### During Clinical Encounters

Participants shared the impact that CGWs can have on provider-patient collaborations and engagement, such as discussing health habits and behavior change.

*Both patients and providers can look at [a step count] and say, I'm tracking a behavior and behaviors or things we can work together to change…let's look at this pattern of your behavior and then you can do something about it. (Physician)*

Participants noted that some subjective measurements can be obtained by self-report (e.g., physical activity, sleep duration), but objective measurements are more useful. Benefits cited include improved data over subjective reports (e.g., avoid recall bias), and objectively collected PGHD can complement subjectively collected self-reported outcomes data (e.g., stress and anxiety perceptions complement heart rate data).

Some CGWs can collect a combination of biometric data that can contextualize clinical data, patients’ health status, or behaviors. HCPs mentioned continuous glucose monitoring data combined with diet and physical activity data with pain perception. The ability to contextualize biometric data in these ways can amplify the usefulness of PGHD to inform treatment and provide signals that the treatment is efficacious.

##### Outside of Clinical Encounters

CGWs can also play a role in patient engagement outside of clinical encounters (Table 3) and can be a valuable complement to a healthy lifestyle. A wellness coach said, “Bringing in the [device] and looking at it with us would [help] them decide if they want to change their goal or make a different goal…We work with them about their goals and their health care.”

**Table 3:** Applications for CGWs Outside of the Clinical Encounter Shared by HCPs during Interviews

Wearable’s function	Illustrative quotes	Real-world case studies or references to existing health system initiatives
Just-in-time prompts^50^	“The [device] is accountability for the [patient]…it’s like a reminder to get up to walk to get up and move….The watch reminds them to take a moment to be mindful when they have pain…it complements other pain management tools in that way.” (Social Worker)	Apple Watch sends user a “you can still do it” nudge if they are falling behind pace to reach daily movement goal
The quantified self^51^	“I think most of them mainly use it for…here’s my oxygen going down, here’s my heartrate going really high. How many hours of sleep am I doing and how many steps? How many miles am I doing?” (Physician)	Fitness watch faces allow swiping between different metrics
Biometric feedback that is normally assessed subjectively	“Because you can change the settings and measure different things…you could do to stress management and log how you’re feeling every day. Check your mindfulness, wear your Fitbit to bed for sleep, so there’s a lot of things.” (Wellness Coach)	Fitbit reports sleep (time per night) and stress levels (heart rate variability) whereas standard medical practice involves a written diary of these metrics
“Wakeup call” that biometrics could provide beyond subjective provider counseling on health risks	“[Continuous glucose monitor users], their awareness portion goes through the roof and they’re like, oh, I really need to make a change. After they passed that, I’m dieting. And it’s easier because they now understand that they really need to take care of this issue.” (Dietitian)	American Association of Clinical Endocrinology has a standard protocol for physicians to discuss continuous glucose monitor data with a patient
Access to momentary and retrospective data	“To be able to use that and look at that and see what their steps were for the week and also see what their sleep averages were…So it’s been very useful to them because it actually gives them a reading of where they may need to make improvements.” (Wellness Coach)	Garmin Connect provides dashboard of summary metrics on phone app or computer desktop web browser

#### Theme 2. Barriers to Utilizing PGHD and CGW

##### Staffing/Workload Issues

Some clinical care providers cited workload issues as a concern about how PGHD would be integrated into clinical workflow. One physician noted that staff is already stretched thin. “Our nurse coordinator…had to answer all [device-related questions], and she needs to be dealing with clinical things.” Another physician mentioned, “We have to balance [the PGHD availability] with our staffing…additional elements that you can do in screening, or turning on or off those features, depending on context of care.”

While some HCPs were enthusiastic about the potential benefits of using PGHD, clinical care providers relayed their concerns over the possibility of receiving automated alerts and preferred to be in control of when and how the data would be used. Such alerts were viewed as tolerable by some interviewees under the condition the alerts were restricted to metrics verified as clinically relevant by a competent third party.

*There has to be some kind of safeguards built-in because I’m not gonna just check that platform every day for fun…If a patient captures their own data and something funny is on it, and they decide to discard it or not pay attention to it, there’s no medical legal liability for the [institution] or for me…But if it’s sitting in a portal somewhere for me, then there is responsibility. (Physician)*

Another physician said they would be willing to review PGHD with patients face-to-face or as part of a telehealth visit, but “[if] something is alerting me…there’s some data [from a device] to be looked at…I don’t think I would want that…Because I already have too much [going on]…” Another physician explained, “When I have an appointment with the patient, I bring [their data] up, [and say] ‘…Let’s look and see what your data looks like’…but I need to be the one that initiates this.”

##### Data Interpretation and Expectations

Some HCPs expressed their confusion over what to do with PGHD and how to discuss the data with their patients.

*The data elements themselves have been in some ways created by people who are not looking to diagnose…as physicians, it can feel a little stuck…the patient [is] coming to me for help… I want to help.,, yet I'm not sure what to do with this [data]. (Physician)*

Interviewees also voiced their concerns about device manufacturers constructing metrics that are often created using proprietary algorithms. Both clinical care providers and holistic/wellness providers mentioned CGWs’ basic biometrics (e.g., steps) could be incorporated into clinical care, but clinical care providers cautioned against the “synthetic indices” (e.g., sleep score) that are built by proprietary algorithms that were never designed for diagnostic purposes. Interviewees advocated for institutional guidance.

*I don't wanna see weird pseudoscience...[or] summary data elements that I don't even know what goes into them. (Physician)*

Another respondent spoke to the data utility.

*Some of these devices, where they bring in a lot of data that may not [carry] a whole lot of meaning to the condition. (Physician)*

Participants also expressed their concern regarding expectations to work with PGHD, as some patients may expect their providers to validate or act based on the PGHD, regardless of the data’s clinical meaning or usefulness. A physician said, “Some patients really do focus on those numbers, and it can sometimes be hard to pull them away from a number that isn’t in the norm….that somehow they’re not getting a gold star on everything from their [device].”

*There are multiple layers of safety when I order a test [through the lab]…there's an automated alert that not only tells me when it’s done; there's even more of an alert if ‘it’s a really abnormal value. None of that happens with consumer [PGHD]. There’s no mechanism there for safety. That puts the physician in a really tough spot. (Physician)*

##### Privacy

Data privacy concerns were discussed in several interviews. A physician mentioned patients often have unaddressed concerns about where their PGHD goes or who will access the data. One wellness coach shared that there was “definitely the concern that you had to share your location”; some HCPs struggled with patients’ questions that have not been clarified by a device manufacturer’s privacy policies.

##### Education

Several participants discussed the need for PGHD competency for patients and HCPs. One physician noted, “… [W]e need to have a way to think about what this data means…it’s not [a] lab test… what does it mean…how do we think about it?.” Another physician discussed the challenge of how to effectively use the data in practice: “…[T]his device is telling [the patient that s/he is not] overachieving in whatever area…and we may or may not have something objective clinically to connect that to.”

#### Theme 3: Suggestions for Integration of CGWs and PGHD into Patient Care

Table 4 contains response to the “ruler-for-the-day” question about an ideal process to integrate CGWs and PGHD into clinical care. The quotes are mapped to the enabling factors of an LHS; Appendix IV contains unabridged quotes.

**Table 4:** A “Ruler-for-the-Day” Wish List of Desired Characteristics or Features of an Ideal Program Involving PGHD from CGWs Mapped to the Enabling Factors of an LHS^26^

Enabling factor	Abridged quotes
The organization has a critical mass of employees with the skills and knowledge necessary for LHS work	Start with the employees first…we can change a culture if we start at the employee level (Physician)
	Buy-in from the providers…educate clinicians (Physician)
Data systems, informatics technology, and resources are in place within the organization to support analyses of clinical data that address the organization’s learning questions	Smoothly transition PGHD into a clinical workflow when appropriate…invest in the IT support and the digital navigation coaches to support the digital literacy gap (Physician)
	Easily accessible for Veterans and HCPs… tying visualizations to goals mutually agreed upon in the shared decision making process between a patient and a clinician (Psychologist)
	Something that’s easy for older patients to import data (Wellness Coach)
	Dashboard integrated directly into the EHR that laid out…various metrics that would be related to each other… annotate and display PGHD in the context of life events and medical treatment (Physician)
	Not very intensive and work intensive for the patient (Nurse)
	Efficient workflow for HCPs…automated on the patient side…buy-in from both sides…friendly interface (Nurse)
	PGHD would be updated every day to medical charts… HCPs can see exactly what patients see…data used to develop different programs and groups (Recreational Therapist)
	Summary of PGHD from past week automatically sent to clinician the day before an appointment (Physician)
	Seamless program for patients to upload their information (Social Worker)
The organization invests resources that are sufficient to carry out the different bodies of LHS work	Everyone would have a device (Wellness Coach)
	Give everyone a phone (Physician)
	“If I was ruler of the world…I would go at it, Oprah style, and you get a phone” (Physician)
There is a supportive organizational culture with norms, policies, and visible leadership that support LHS work	Share information about all devices available to patients (Wellness Coach)
	Allow staff to have dedicated time to follow up with patients…provide staff with a device (Physician)
	Patient groups can help monitor changes and support each other (Wellness Coach)
	Expand access to programs using devices and evaluate the reach of the programs (Dietitian)
	Have the program that is pretty open that doesn’t have a lot of requirements to participate (Dietitian)
	Comprehensive outpatient approach with pain management, also involves nutrition weight loss (Physician)

##### Clinical Workflow

Participants relayed their perspectives on accessing PGHD and how it might affect clinical workflow. Many clinical care providers had been relying on ad hoc self-reports, such as looking at the patient’s phone screen during the clinic visit or asking the patient to provide screenshots through secure messaging.

*We’ve got all this data, but [patients] really aren’t getting it to their providers, to their nurses… I think [providers] would really enjoy looking at what [patients are] doing and may be able to support them, help them, educate them on some of these parameters that we’re seeing in their data. (Dietitian)*

One physician mentioned that “patients would show up and say, ‘I’ve got this wearable…this is what it’s showing and is this helpful’.” Another physician reported that a patient provided personal login information to the provider to view the patient’s dashboard, which made the provider feel “awkward and intrusive and would prefer a backend dashboard with ongoing sharing capabilities.”

##### User Experience

Participants presented several suggestions related to user experience, such as a dashboard, “minimal keystrokes,” “accessible on demand,” “ability to toggle between data elements,” and “singular interface.” One participant said, “Seamless integration is key.” A physician said, “The fewer places that clinicians have to look for information the more likely it is to be transmitted meaningfully to impact patient care.” While general sentiments favored ease of access to PGHD, other HCPs appeared less concerned with potential additional steps, such as logging into a separate portal to view PGHD.

*If there is some sort of system that could integrate all that data into the VA documentation system, that would be amazing. … if we can have a wearable device …and then if that data could be shared with us… that's usable data … I'd be very interested. (Recreational Therapist)*

Physicians mentioned the need for PGHD guidelines from their institutions, so the information is medically actionable.

*[We need] a committee that says these are the care pathways that are being attributed to these remote monitoring devices or smart devices that have evidence behind them. These are the parameters of what we would consider clinical thresholds by which specified information will be sent over to the electronic health record and thus now it’s HIPAA. (Physician)*

##### Educational and Technical Support

Both clinical care providers and holistic/wellness providers expressed a need for patient support to use CGWs in clinical care, with respect to technical guidance and infrastructure resources (e.g., smartphones, data service).

*I think any future opportunities to bring wearable devices to [patients] need to come with some sort of backend tech support. I don’t think that’s something that clinicians are really going to be capable of managing on top of, you know, a lot of the other responsibilities and reminders and all that kind of stuff… 1-800 [tech support] number or something. (Physician)*

Other supports (e.g., computer access, internet connectivity) are needed to support CGW adoption. Several participants commented that smartphone ownership is an important factor to consider because CGWs often require connection to a smartphone.

*There’s a good amount of research out there showing that those that have smart devices are typically people that are well educated and have financial means to purchase them…From an access and health equity standpoint, there’s a problem because we’re only able to [have] these conversations around…a privileged group… we can give someone a smart device but the real power behind its full impact on behavior change is tethered to a smartphone. (Physician)*

Interviewees also stated that technical and educational support should be provided as a system-level “directive” spearheaded by leadership.

## DISCUSSION

The ability to reliably access, critically assess, and strategically analyze PGHD and apply findings to inform continuous improvement of care delivery is a central tenet of an LHS. We elicited the perspectives of HCPs from a large integrated health system on the utility of CGWs and the types of support needed to integrate PGHD into their clinical workflow. HCPs were generally enthusiastic about the potential of CGWs to help engage and communicate with patients, but also had concerns about feasibility. Participants called for support for patients (e.g., physical resources, technical support) and HCPs (e.g., integrated dashboard). A few interviewees added that efforts to integrate CGWs and PGHD must consider health equity since patients experiencing the digital divide typically have limited access to quality care.^25^

Several participants spoke about the need for a streamlined approach to access PGHD and their concerns about expectations to review PGHD that they do not understand or consider clinically actionable. Concerns about increased cognitive load may reflect infrastructure constraints (e.g., understaffing) that reduce the amount of time an individual HCP can cogitate upon and apply PGHD. Interviewees advocated for a coordinated strategy from their institution (or the wider medical community) to provide guidance on which metrics can and should inform clinical care, as well education on how to use PGHD—for patients and HCPs—to ensure users understand the benefits and limits of PGHD.

### HCP Perspectives May Vary by Job Role

An LHS engages HCPs “in the process of learning, knowledge generation, and translation”^24^; thus, having HCP buy-in is fundamental. A novel aspect of this study was disaggregating responses by job role, as clinical care providers and holistic/wellness providers have different decision-making authority guiding their work. Both groups were enthusiastic about the impact PGHD had on improving patient communication and engagement. However, opinions varied on how and when to use PGHD.^26,27^

Clinical care providers were concerned about the potential patient safety issues of unmonitored PGHD, the appropriate applications of PGHD in clinical care,^28^ and workload issues tied to expectations of interpreting PGHD they did not “order.”^29^ Along similar lines, the existing definitions of what constitutes a symptom of malaise are challenged by a patient’s PGHD that might suggest the presence of disease or an imminent health-related adverse event without a patient feeling ill, which could complicate the provider-patient interaction.^30–32^ Conversely, holistic/wellness providers relied more on PGHD to provide information about patient behaviors and using this information to motivate patients to make behavioral changes. Their contrasting perspectives highlight the various perceived applications of PGHD from CGWs based on HCP job roles and the nature of their interactions with patients. Therefore, a one-size-fits-all approach to integrate CGWs and PGHD into clinical workflow is counterintuitive, underscoring the importance of including various HCP perspectives.^29,33^

### Previous Work

Our findings echo the perspectives from other qualitative studies of interviews with HCPs about CGWs and PGHD.^4,5,9,34^ Saleem et al.^35^ interviewed VHA clinicians and had reported similar perceived uses of a smartwatch (e.g., promoting engagement in patients’ self-management outside of clinical appointments). Our findings mirrored the themes identified in a scoping review^34^ about how CGWs and PGHD could empower patients and barriers to CGW use. Other researchers had highlighted HCPs’ increasing interest in using PGHD in clinical settings but were cautious about what data elements should be used to complement traditional clinical data.^36^ Our interviewees further echoed reports of limited adoption of PGHD in small pilots or short-term interventions, often in specific settings, providing evidence that PGHD has yet to be integrated into routine clinical workflow.^37^ This hesitancy could be attributed to many factors also raised by our interviewees, including the need for education on how to use PGHD,^38^ potential for PGHD to impact “digital fatigue” and burnout among providers whose workload is already overburdened,^39^ and support needed to help patients use CGWs^40^ and PGHD. Incentivizing and/or promoting PGHD integration through targeted implementation efforts may ameliorate these concerns.^29,36,41^

### Integrating CGWs and PGHD into Clinical Care to Enable an LHS

Findings from our study extend previous work by including not only the perspectives of clinicians but other HCPs such as wellness coaches. We also synthesized findings into a discussion of how integrating CGWs and PGHD into clinical care can enable an LHS by mapping responses from the “ruler-for-the-day” question to the LHS-CF.^24^ These suggestions underscore the importance of cultivating expertise, having resources and infrastructure in place to support data analysis and synthesis, investing resources to enable collaboration between units, and building a supportive organizational culture when developing a comprehensive and coordinated strategy to integrate CGWs and PGHD into clinical care. This will enable and support an LHS’ ability to collect and analyze data to generate insights that are then combined with other experience and evidence into practice to improve care delivery and patient outcomes.

Integrating CGWs and PGHD into clinical workflow may adversely affect access and quality of care and exacerbate health inequities^30^ because individuals experiencing the digital divide^42,43^ may not be able to fully participate in CGW and PGHD interventions.^26,27,44^ As achieving health equity is becoming “a critical goal” of an LHS,^24^ there is potential for an LHS to attenuate or eliminate the negative impacts of utilizing CGWs and PGHD and improve access and health equity.

### Limitations

First, as interviews were conducted with HCPs who work at VHA, findings may not be applicable to other health systems. However, VHA is the largest integrated health system in the USA and is an important testing ground for the pitfalls and opportunities of CGWs and PGHD. Second, although we sampled participants purposively and had a high response rate, our results do not necessarily reflect the views of all HCPs that have experience with PGHD or CGWs. Our sample did include HCPs with varied job roles, training, and years in practice, albeit a small number of holistic/wellness providers. Thirdly, the purposive sampling strategy of participants of a CGW distribution program excluded patients and caregivers. Research to elucidate HCP perspectives should be paired with patient perspectives to understand the patient-HCP dyad as they navigate opportunities and challenges related to the use of CGWs and PGHD.

### Future Directions and Considerations

CGWs are commercial products and device manufacturers have largely driven the conversation about accessing, viewing, and using PGHD through their mobile apps’ visual metrics created by proprietary algorithms that might not be clinically relevant. The gamification of “health” and “wellness” (e.g., “sleep score,” “body battery”) to motivate behavioral change has further complicated the interpretation of PGHD from CGWs. Often, a requisite to setting up and using a CGW is for the consumer to agree to the manufacturer’s terms and conditions. While a device manufacturer may declare they do not sell data, they can and do share data with an unrestricted list of partners to be used for sales, marketing, research, and data analysis.^45^

Health systems can gain some control over access to and use of PGHD by determining which metrics have clinical value and making only those PGHD visible to patients and HCPs, while also providing guidance on how this PGHD should be used to inform clinical care. An LHS should include prioritizing data use policies and data security and privacy as part of its organizational culture to build trust with HCPs and patients, particularly in present day scenarios where biometrics may be used to develop artificial intelligence algorithms, all of which could be detrimental to a health system’s commitment to health equity.^29^ Furthermore, there are legal liability issues for clinicians regarding PGHD interpretation because PGHD are high volume (i.e., CGWs generate millions of data points per day), and are usually not ordered by the provider (i.e., patients bring their own CGWs). This requires clear guidelines from health systems to help set expectations for clinician responsibility.

It is evident from HCP perspectives that integrating CGWs and PGHD into clinical care will require a system redesign—what is the work, who will do the work, what tools will be used to perform the work, and how to do the work. Findings from this study provided insights to high-level as well as practical considerations and solutions that could inform the development of a roadmap for health systems to integrate CGWs effectively. This should include assembling a stakeholder committee of patient and caregivers in addition to HCPs and other personnel with various job roles charged with developing solutions to address specific needs (Table 5).

**Table 5:** Professionals to Include in a Stakeholder Committee

Example of solution or consideration	Personnel
CGW dashboard (integration into EHR, toggling between data elements)	Software engineers, data analysts
AI-driven “flagging” of clinically significant deviations to reduce HCP burden and cognitive load (e.g., remote hypertension monitoring)	Research scientists and associates with expertise in developing AI algorithms
Types of reimbursement models that might incentivize PGHD use	Hospital finance, billing professionals
Protocol to pragmatically control the legal liability for clinicians regarding PGHD interpretation	Lawyers, digital ethicists
Establishing guidelines for what data elements to include	Healthcare professionals (e.g., primary care physicians, specialty care physicians, nurses, research scientists)

## CONCLUSIONS

This mixed methods study focused on exploring the clinical potential of CGWs and PGHD in a purposive sample of HCPs at the largest healthcare system in the USA. Most participants were enthusiastic and provided insight into strategies to overcome feasibility considerations, but also voiced concerns surrounding the efficient and ethical management of PGHD. Increased attention should be placed on clinical workflow changes to minimize HCP burnout and fatigue. These findings convey an urgency to build technical infrastructure to support PGHD access, and include technical support for devices and education around the benefits and limits of PGHD, institutional guidelines for appropriate use of PGHD, and strategies to optimize workflow, to benefit patients and HCPs. A coordinated strategy to integrate CGWs and PGHD into clinical care can enable an LHS, which will support an LHS’ ability to collect and analyze data to generate insights to continuously improve care delivery and patient outcomes.

## Supplementary Information

Below is the link to the electronic supplementary material.

Supplementary file1 (DOCX 36 KB)

## Data Availability

The datasets during and/or analyzed during the current study are available from the corresponding author on reasonable request.
