# Supplementary material for: Integrating Consumer-Grade Wearable Devices and Patient-Generated Health Data into Clinical Care: Perspectives from Healthcare Professionals at a Learning Health System
Source: J Gen Intern Med. Author manuscript; Available in PMC 2026 Mar 3. (PMC12954729; doi:10.1007/s11606-025-09876-x)
Supplement: Supplement [file NIHMS2138612-supplement-Supplement.docx]

**Appendix I. COREQ (Consolidated Criteria for Reporting Qualitative Studies (COREQ): 32-Item) Checklist**

| **Item No.** | | **Guide Questions/Description** | **Reported on Page #** |  |
| --- | --- | --- | --- | --- |
| **Domain 1: Research team and reflexivity** | | | |  |
| **Personal Characteristics** | | | |  |
| 1. Interviewer/ facilitator | | Which author/s conducted the interview or focus group? | Pg 9 |  |
| 2. Credentials | | What were the researcher’s credentials? E.g., PhD, MD | Pg 9 |  |
| 3. Occupation | | What was their occupation at the time of the study? | Pg 9 |  |
| 4. Gender | | Was the researcher male or female? | Pg 9 |  |
| 5. Experience and training | | What experience or training did the researcher have? | Pg 9 |  |
| **Relationship with participants** | | | |  |
| 6. Relationship established | | Was a relationship established prior to study commencement? | Pg 9 |  |
| 7. Participant knowledge of the interviewer | | What did the participants know about the researcher? e.g. personal goals, reasons for doing the research? | Pg 9 |  |
| 8. Interviewer characteristics | | What characteristics were reported about the interviewer/facilitator? e.g. Bias, assumptions, reasons and interests in the research topic | Pg 9 |  |
| **Domain 2: study design** | | |  |  |
| **Theoretical framework** | | |  |  |
| 9. Methodological orientation and Theory | What methodological orientation was stated to underpin the study? e.g. grounded theory, discourse analysis, ethnography, phenomenology, content analysis | Pg |  |  |
| **Participant selection** | | |  |  |
| 10. Sampling | How were participants selected? e.g., purposive, convenience, consecutive, snowball | Pg 8 |  |  |
| 11. Method of approach | How were participants approached? e.g., face-to-face, telephone, mail, email | Pg 8 |  |  |
| 12. Sample size | How many participants were in the study? | Pg 8 |  |  |
| 13. Non-participation Setting | How many people refused to participate or dropped out? Reasons? | Pg 8 |  |  |
| 14. Setting of data collection | Where was the data collected? e.g., home, clinic, workplace | Pg 8 |  |  |
| 15. Presence of nonparticipants | Was anyone else present besides the participants and researchers? | N/A |  |  |
| 16. Description of sample | What are the important characteristics of the sample? e.g. demographic data, date | Pg 10 |  |  |
| **Data collection** | | |  | No |
| 17. Interview guide | Were questions, prompts, and guides provided by the authors? Was it pilot tested? | Pg 9 |  |  |
| 18. Repeat interviews | Were repeat interviews carried out? If yes, how many? | N/A |  |  |
| 19. Audio/visual recording | Did the research use audio or visual recording to collect the data? | Pg 9 |  |  |
| 20. Field notes | Were field notes made during and/or after the interview or focus group? | Pg 10 |  |  |
| 21. Duration | What was the duration of the interviews or focus group? | Pg 10 |  |  |
| 22. Data saturation | Was data saturation discussed? | N/A |  |  |
| 23. Transcripts returned | Were transcripts returned to participants for comment and/or correction? | N/A |  |  |
| **Domain 3: analysis and findings** | | |  |  |
| **Data analysis** | | |  |  |
| 24. Number of data coders | How many data coders coded the data? | Pg 10 |  |  |
| 25. Description of the coding tree | Did the authors provide a description of the coding tree? | N/A |  |  |
| 26. Derivation of themes | Were themes identified in advance or derived from the data? | Pg 10 |  |  |
| 27. Software | What software, if applicable, was used to manage the data? | Pg 10 |  |  |
| 28. Participant checking | Did participants provide feedback on the findings? | N/A |  |  |
| **Reporting** | | |  |  |
| 29. Quotations presented | Were participant quotations presented to illustrate the themes/findings? Was each quotation identified? e.g., participant number | Pg 10-20 |  |  |
| 30. Data and findings consistent | Was there consistency between the data presented and the findings? | Pg 10-20 |  |  |
| 31. Clarity of major themes | Were major themes clearly presented in the findings? | Pg 10-20 |  |  |
| 32. Clarity of minor themes | Is there a description of diverse cases or a discussion of minor themes? | Pg 10-20 |  |  |

**Appendix II. Interview Guide**

Before we begin, I’d like to clarify that when I mention a wearable device, I am referring to a device worn on the body that can sense the person who wears them and/or their environment. “Consumer-grade wearable devices” are the ones intended for general wellness use, unrelated to diagnosing or treating a chronic disease. They carry low risk of creating a medical complication if they malfunction. Some examples are the Fitbit, Apple Watch, Oura Ring, and Whoop.

1. Please tell me about your experience with using consumer-grade wearable devices in patient care, beginning with how you started using them in your current position.

2. What are some of the things your facility has implemented related to use and/or interest in using patient-generated data as part of clinical workflow?

a. Probe: Current barriers/friction points

3. Suppose you were ruler for the day, and could make all the changes you needed to set up a program to integrate patient-generated data into clinical workflow, what would that look like today?

a. What’s your vision for the changes 3 years from now?

4. Is there anything else that you think is important that we haven’t already discussed?

**Appendix III. Illustrative Quotes by Theme**

| **Theme** | **Illustrative Quotes** |
| --- | --- |
| **Use and Utility of CGWs and PGHD** | “[Making] some sort of connection between goals and the use of wearable devices … I do feel like that's where it was most useful, and people were the most engaged and then most likely to benefit from having that additional technology.” (Physician) |
|  | “[Many patients are] already motivated and have a sense of a goal and we use [the device] as a resource to help them reach that goal.” (Social Worker) |
|  | “If the [health system] asks [patients] to hook up data, there would be expectation that the [health system] will do something with that data. If there is no plan for the data, it will look like the [health system] is not providing care.” (Physician) |
|  | “Typically, that two-week time is going to give me enough variation of what [the] [patient] is [doing]… [A patient] … newly diagnosed with diabetes maybe just on a few [medications], we're going to be able to help them with those areas. I'm going to look for blood sugar spikes on their [continuous glucose monitors]. For some [patients] that they need, they need to see how … food and exercise impacts them. We have done continuous glucose monitoring in our primary clinic for four years and it's just been a really, really great program.” (Dietitian) |
|  | “It was hard to get people to do homework on a regular basis…having a product to track some of the things…would be helpful…[asking] how [did] things go this past week… there might be a recall bias if you try to ask it retroactively.” (Social Worker) |
|  | “…the various metrics that would be related to each other…I keep coming back to sleep and daily activity…we could kind of layer…the stress metric [and] periods of elevated heart rate during low activity…on a single dashboard…[or] across time.” (Physician) |
|  | “They noticed when their heart rate goes up, maybe in a stressful situation because we also work with them with guided imagery in this station, so they were able to monitor their stress through that as well.” (Wellness Coach) |
|  | “Before this, I don’t think I had any [experience with consumer grade wearable devices]. I did notice that I referred some people for pulmonary rehabilitation and a couple of patients who used their [consumer grade wearable device], I think it just went better for them. A lot of that is our pulmonary rehab is all virtual. So there’s a lot of telephone calls. The patient reporting is difficult, whereas someone who had like a Fitbit could say I was walking 8000 steps a day. I did 2.5 miles and heart rate went up to a specific amount. So it was a lot more helpful for the person leading them in rehabilitation. They just had better data.” (Physician) |
|  | “Anything that can be recorded that is not something that the patient has to report is only going to help you more.” (Dietician) |
|  | “The watch reminds them to take a moment to be mindful when they have pain. So it complements other pain management tools in that way.” (Social Worker) |
|  | “Integrating wearable data into [a patient’s] dashboard so that we can look at that data side by side with the patient reported outcomes. [Clinicians] could look at the data themselves again alongside the patient reported outcomes like [the nine-item Patient Health Questionnaire for depressive symptoms] to see how that data correlates.” (Physician) |
| **Barriers to Utilizing CGW and PGHD** | “…the various metrics that would be related to each other…I keep coming back to sleep and daily activity…we could kind of layer…the stress metric [and] periods of elevated heart rate during low activity…on a single dashboard…[or] across time.” (Physician) |
|  | “They noticed when their heart rate goes up, maybe in a stressful situation because we also work with them with guided imagery in this station, so they were able to monitor their stress through that as well.” (Wellness Coach) |
|  | “Before this, I don’t think I had any [experience with consumer grade wearable devices]. I did notice that I referred some people for pulmonary rehabilitation and a couple of patients who used their [consumer grade wearable device], I think it just went better for them. A lot of that is our pulmonary rehab is all virtual. So there’s a lot of telephone calls. The patient reporting is difficult, whereas someone who had like a Fitbit could say I was walking 8000 steps a day. I did 2.5 miles and heart rate went up to a specific amount. So it was a lot more helpful for the person leading them in rehabilitation. They just had better data.” (Physician) |
|  | “Anything that can be recorded that is not something that the patient has to report is only going to help you more.” (Dietician) |
|  | “The watch reminds them to take a moment to be mindful when they have pain. So it complements other pain management tools in that way.” (Social Worker) |
|  | “We don't like to order lab tests that we don't know what to do with…so the convention is anything you order or any data you collect is telling you something that's gonna help make a decision.” (Physician) |
|  | “It's not just I don't want to order something I'm not responsible for. There's the potential to put someone at risk because we find a signal [from the PGHD] that we don't completely understand.” (Physician) |
|  | “Pseudo-scientific medical data creates another layer of responsibility—who is monitoring that data…” (Physician) |
|  | “Overall, a major issue is getting complete data. About everything, not just what's tracked by the Fitbit. Things that can't be passively monitored and require active monitoring like food and pain. Especially older Veterans who may be challenged using the technology, or forgetful about logging.” (Social Worker) |
| **Suggestions for Integration of CGWs and PGHD into Patient Care** | “I think there's always interest in these new things and offering things to [patients]…there's so much effort put into building [new things] and to creating them and very little to sustaining them…If [our institution] decides to partner with [a device manufacturer]…it's a consumer thing we are in some ways giving our approval of it and I think that now we're really on the hook to make sure that we've thought of all of the potential negatives.” (Physician) |
|  | “I have occasionally with individual [patients] like looked at that on their device with them. But we've not consistently had like a good strategy for how to like import that… there have been conversations with other team members about ways that that could be sent through…secure [messaging] but we certainly don't have a consistent way in which we're either receiving [or] documenting that information beyond self-report.” (Physician) |
|  | “It would be optimal to have, you know, a singular interface, but again if there were another secure or function like a dashboard… fewer clicks. The batter and the fewer places that clinicians in particular, have to look for information. The more likely. It is to be transmitted meaningfully to impact patient care.” (Physician) |

**Appendix IV. A “Ruler-for-the-Day” Wish List of Desired Characteristics of an Ideal Program Involving PGHD from CGWs Mapped to the Enabling Factors of an LHS**

| **Enabling Factor** | **Illustrative Quotes** |
| --- | --- |
| The organization has a critical mass of employees with the skills and knowledge necessary for LHS work. | **“**We would have started with the employees first because as we are trying to change a culture if we change the culture at the employee level. Then the next step is going to happen almost naturally, it's just gonna flow.” (Physician) |
|  | **“**Buy-in from the providers. You have to educate [the] clinician.**”** (Physician) |
| Data systems, informatics technology, and resources are in place within the organization to support analyses of clinical data that address the organization's learning questions. | **“**We have to integrate this information, we have to smoothly transition it into a clinical workflow when appropriate. [It means] we have to invest in the IT support and the digital navigation coaches to support the digital literacy gap that exists.**”** (Physician) |
|  | **“**Easily accessible on both ends, so both in terms of how Veterans are sharing that information as well as how clinicians are receiving it so some type of system in which it's like an easy button…visualizing data would also be something that would be important for it to have a meaningful clinical impact… I've often found myself…looking at weight and tracking weight over time and how having that graph with timepoints people really respond to that in a clinical environment, so I think something that offered visualization would be really important, and then tying it…to goals.**”** (Clinical Psychologist) |
|  | **“**I always try to think of a fix, a easier way, even for them. The older … older patients so they can feel like they could be a part of it, trying to waiting something that's easy to use where they can import data.**”** (Wellness Coach) |
|  | **“**Dashboard integrated directly into the EHR that laid out, you know, the various metrics that would be related to each other.**”** (Physician) |
|  | “[The program would not be] very time intensive and work intensive for the [patient].”(Nurse) |
|  | **“**You need to make your workflow efficient, or else I'll never get by and so that's the first thing we need. But it needs to be automated on the [patient] side too… it's going to have a computer to type their data into the VA is very difficult. So we need Bluetooth capabilities. So automation, Bluetooth capability with we get buy-in from both sides and we get a user friendly interface.**”** (Nurse) |
|  | **“**We would see them and just that data would get directly updated every day to their medical chart and we can see exactly like what they see we see, especially like I said regarding the sleep and the movement, those are the two really big pieces that help us or help me. And that would be pretty fantastic. And then this way we also know how to like cater and check and then we can kind of come up with different programs and groups. [maybe also the 1st feature].**”** (Recreational Therapist) |
|  | **“**[PGHD] would automatically get sent to the summary of that would get sent to the clinician the day before the appointment of summary of the past week.**”** (Physician) |
|  | **“**Visualizing data would also be something that would be important for it to have a meaningful clinical impact…Some ability to tie it to goals…that data also has to be linked to something meaningful and anchored in you know a shared goal between the clinical team or provider and with the veteran, whether that is some other biometrics and we've spoken mostly about some of the things that are accessible through Fitbit. But even you know A1c or blood pressure or some of that other biometric data if it could be anchored. Tying to a stated goal that's mutually agreed upon in the shared decision making process between a patient and a clinician. That's where I think it would be most useful.**”** (Clinical Psychologist) |
|  | “[I] guess I'm also trying to think of like veteran engagement and then initiative and kind of how their reporting the information…I think you know if there was a huge way for them to upload their information, that would be sort of in the magic wand world. That would be a little bit more seamless instead of relying on some, you know, soft report of the of the information.**”** (Social Worker) |
|  | **“**So you're watching kind of how things evolved is. Patient reported outcomes in terms of mood and anxiety and annotations related to starting or stopping any intervention medications. For example, we started ahead, we stopped a Med, we started psychotherapy. We stopped psychotherapy. Uh, so medical stuff and life events, you know, lost a job, got a new job, bought a house, you know, child born, bought a new, you know, got a new dog. Got a new pet. Whatever. So, you know, annotating kind of life events and that sort of thing. And so these it is a easily digestible, you know, you know. display that is using, putting that wearable data Sleep and activity in the context of life events and medical treatment.” (Physician) |
| The organization invests resources that are sufficient to carry out the different bodies of LHS work. | **“…e**veryone would have a device.**”** (Wellness Coach) |
|  | **“…** give everyone a phone**…”** (Physician) |
|  | **“**And [in a] perfect world, I would give every single [patient] a smartphone where we pay their cellular service... We have smartphones available that we will hand to them when they come to the hospital or clinic as a loaner. The smartphone device and patient-generated data devices are tools that we need to invest in as the new generation of how medicine operates and as tools of our medical profession. We have not gotten there yet, but if I was ruler of the world, you know, I would go at it, Oprah style, and you get a phone.**”** (Physician) |
| There is a supportive organizational culture with norms, policies, and visible leadership that support LHS work. | **“**I think that I would make everything known that's available to the Veterans. I would make it known because there's so many things that are available to them that they have no idea and some of them have been in the system for a very long time.**”** .**”** (Wellness Coach) |
|  | **“**Give that staff a staff member of the time to do it dedicates something so somebody has the time to follow up with the veteran to make the phone calls to send the letters as secure messages to say any problems. Did you get the device answer any questions provide helpline numbers also would have been really helpful if I'd been provided with a Fitbit.**”** (Physician) |
|  | **“**Monitoring their change and I think that the groups are helping with that, too kind of supporting each other too.**”** (Wellness Coach) |
|  | **“**I would love to see is did we help Veterans who weren't involved with us at all…Did we reach a different group of Veterans on by opening it up to other people?**”** (Dietitian) |
|  | **“…**have the program that is pretty open that doesn't have a lot of requirements to participate… I'm taking into consideration our [patient] population that has so many social determinants and mental health issues…comprehensive outpatient approach [with] pain management…also involves nutrition weight loss.**”** (Dietitian) |

| **Appendix V-a. Survey Responses of VHA Clinical Care* Providers (n = 15)** | | | |
| --- | --- | --- | --- |
| **Survey items** | **Agree or Strongly Agree** | | **Mean (SD) out of 5** |
| **Interest in uses of CGWs at clinical encounters**  (1=strongly disagree, 2=disagree, 3=neither agree or disagree  4=agree, 5=strongly agree) | | | |
| CGWs can promote engagement between the health care provider and patient or caregiver. | 14 (93.3) | 4.1 (0.5) | |
| In the future, if my patient wore a sensor that automatically collected information about their health or well-being, I would refer to the data to understand how my patient is responding to treatment. | 15 (100.0) | 4.5 (0.5) | |
| I would be willing to try out a new CGW technology in my practice before it had been clinically validated. | 14 (93.3) | 4.4 (0.6) | |
| CGWs may provide me with a way to communicate my patient’s progress more clearly. | 14 (93.3) | 4.3 (0.6) | |
| I would find it useful to use CGWs to monitor my patient’s progress (e.g., using a Fitbit to assess their daily activity levels). | 14 (93.3) | 4.4 (0.6) | |
| **Interest in CGWs outside of clinical encounters** | | | |
| Consumer-grade wearable devices may help my patients stay engaged (especially outside our face-to-face sessions) | 15 (100.0) | 4.3 (0.5) | |
| **Interest in add-ins** | | | |
| I would like consumer-grade wearable devices to interface with mobile video- and text-based coaching. | 13 (86.7) | 4.5 (0.7) | |
| I would like consumer-grade wearable devices to interface with mobile instructional videos (e.g., Fitbit Premium). | 15 (100.0) | 4.3 (0.5) | |
| **Feasibility** | | | |
| I expect learning how to use a CGW in a way that helps my patients will be quite difficult for me. | 2 (13.3) | 2.3 (1.1) | |
| I expect it will be difficult to teach or coach my patients on the use of a CGW (e.g., Fitbit, Oura Ring™). | 4 (26.7) | 2.8 (0.9) | |
| I expect using a CGW in my practice will take a lot of extra time. | 3 (20.0) | 3.1 (1.0) | |
| I would be willing to use a new CGW technology if I received adequate support. | 14 (93.3) | 4.3 (0.6) | |
| Please elaborate on adequate support (Open response):   - Technical support for patients - Digital navigators - Active follow-up to prevent patients from passively abandoning device - Improved methods to share data smoothly and securely - Digital technology coach to train patients - A direct help line for patients - Clearly written user guide - Online platform to ask questions - Customer service education - Troubleshooting assistance to offload level of help needed from the institution - Tech support line - YouTube video tech support for patients - Live patient level support | | | |

*: Physicians, nurses, dietitians, social workers.

The frequency of Agreed/Strongly Agreed survey responses is presented as n (%)

| **Appendix V-b. Survey Responses of VHA Holistic/Wellness† Providers (n = 6)** | | | |
| --- | --- | --- | --- |
| **Survey items** | **Agree or Strongly Agree** | | **Mean (SD) out of 5** |
| **Interest in uses of CGWs at clinical encounters**  (1=strongly disagree, 2=disagree, 3=neither agree or disagree  4=agree, 5=strongly agree) | | | |
| CGWs can promote engagement between the health care provider and patient or caregiver. | 5 (83.3) | 4.2 (0.8) | |
| In the future, if my patient wore a sensor that automatically collected information about their health or well-being, I would refer to the data to understand how my patient is responding to treatment. | 4 (66.7) | 4.2 (1.0) | |
| I would be willing to try out a new CGW technology in my practice before it had been clinically validated. | 4 (66.7) | 4.0 (0.9) | |
| CGWs may provide me with a way to communicate my patient’s progress more clearly. | 4 (66.7) | 4.0 (0.9) | |
| I would find it useful to use CGWs to monitor my patient’s progress (e.g., using a Fitbit to assess their daily activity levels). | 3 (50.0) | 3.8 (1.0) | |
| **Interest in CGWs outside of clinical encounters** | | | |
| Consumer-grade wearable devices may help my patients stay engaged (especially outside our face-to-face sessions) | 5 (83.3) | 4.2 (0.8) | |
| **Interest in add-ins** | | | |
| I would like consumer-grade wearable devices to interface with mobile video- and text-based coaching. | 5 (83.3) | 4.3 (0.8) | |
| I would like consumer-grade wearable devices to interface with mobile instructional videos (e.g., Fitbit Premium). | 4 (66.7) | 4.2 (1.0) | |
| **Feasibility** | | | |
| I expect learning how to use a CGW in a way that helps my patients will be quite difficult for me. | 0 (0.0) | 1.7 (0.8) | |
| I expect it will be difficult to teach or coach my patients on the use of a CGW (e.g., Fitbit, Oura Ring™). | 0 (0.0) | 2.0 (0.6) | |
| I expect using a CGW in my practice will take a lot of extra time. | 0 (0.0) | 2.0 (0.9) | |
| I would be willing to use a new CGW technology if I received adequate support. | 3 (50.0) | 4.0 (1.1) | |
| Please elaborate on adequate support (Open response):   - Technical support for patients to offload level of help needed from the institution - Education | | | |

†: Wellness coaches and recreational therapist.

The frequency of Agreed/Strongly Agreed survey responses is presented as n (%)
